# Unravelling subjectivity, embodied experience and (taking) psychotropic medication

**DOI:** 10.1016/j.socscimed.2019.04.004

**Published:** 2019-06

**Authors:** Jacinthe Flore, Renata Kokanović, Felicity Callard, Alex Broom, Cameron Duff

**Affiliations:** a Social and Global Studies Centre, RMIT University, Melbourne, Australia; b Monash Centre for Health Research and Implementation, Monash University, Melbourne, Australia; c Birkbeck Institute for Social Research, Birkbeck, University of London, United Kingdom; d Centre for Social Research in Health, UNSW Arts and Social Sciences, The University of New South Wales, Sydney, Australia; e School of Management, RMIT University, Melbourne, Australia

**Keywords:** Mental health, Feminist science studies, Intra-action, Psychotropic medication, Psychiatry, Subjectivity

## Abstract

This paper explores how distinctions between ‘intended’ and ‘side’ effects are troubled in personal narratives of taking psychotropic medications. Grounded in interviews with 29 participants diagnosed with mental illness in Victoria, Australia between February and December 2014, we consider how people interpret pharmaceutical compounds beyond their desired or intended effects, and how such effects shape and transform subjectivity and their relationship with their bodies. This paper contributes to recent discussions of mental illness and medication effects, informed by feminist science studies. It emphasises the co-constitution of social, affective and material relations in the context of ‘taking’ psychotropic medication. This paper discusses three key themes as important to the phenomenology of the nexus of illness and psychotropic medication: movement, ambivalence, and sociality. Our analysis demonstrates how psychotropic drugs are *productive* of subjectivity *through* their promises and potential, their unexpected harms and the institutions from which they are inseparable.

## Highlights

• Analyses accounts of mental illness using feminist science and technology studies.
• Focus on experiences of taking psychotropic drugs and impact on subjectivity.
• Moves beyond distinctions between ‘therapeutic’ and ‘side’ effects of drugs.
• Unique contribution to understandings of experiences of taking psychotropic drugs.

## 1 Introduction

Personal narratives about taking medication reveal the complex, often ambiguous and social dimensions of pharmaceuticals. As technologies with discrete (intended) actions and effects, psychotropic medications, those that target health conditions attracting psychiatric diagnoses, act on bodies and subjectivities (Duff, 2014; Schlosser and Hoffer, 2012). Medications animate organs and subjectivities in multifaceted, unexpected and ambivalent ways as they circulate inside and outside the body. These circulations intersect with other social, affective and material forces as they act on individual bodies, resonating with some, interfering with others, as they enmesh the body in relations of class, gender, power and race (Duff, 2014; Fraser et al., 2014; Roberts, 2014; Blackman, 2012). Varying according to these relations, the effects of psychotropic medications differ considerably between persons (Goldberg and Ernst, 2012), and while they can potentially minimise symptoms associated with mental illness, they are known to cause bodily changes such as temperature fluctuations, weight gain and blurred vision (Keltner and Folks, 2005). Psychotropic medications are a class of pharmaceuticals intended to act on individuals' mood, perception and behaviour, inevitably shaping subjective experience in ways that invite greater scrutiny into their impacts on subjectivity and embodiment (Fraser et al., 2014; Duff, 2013). These medications act on the central nervous system to ‘balance’ the brain's neurochemistry, producing effects that shape how they have become known, for example as mood stabilisers, tranquilisers or antipsychotics (Keltner and Folks, 2005). Whilst often less emphasised within the scholarly literature and certainly in clinical contexts (e.g. Goldberg and Ernst, 2012; Keltner and Folks, 2005), these medications have an equally marked, but also socially and materially varied, effect on identity and subjective experience, often transforming individuals' agency, relations, mood, habits and temperament (Moncrieff, 2009).

Instead of approaching indicated effects or unintended side effects of psychotropic medications as separate phenomena, this paper explores how such distinctions are troubled. Grounded in original empirical research conducted in Victoria, Australia, we consider how people interpret and understand the actions of pharmaceutical compounds beyond their intended effects, and how such effects shape and transform participants' subjectivity and their relationship with their bodies. This paper contributes to recent discussions of mental illness, treatment and medication effects informed by feminist sciences studies, particularly the work of Karen Barad (2007), to demonstrate the complex ‘intra-actions’ by which treatment effects, subjectivities and embodied experience are co-constituted in complex social, affective and material relations. We take Barad's work, and feminist science studies more broadly, as a means of troubling the relationships between medication, subjectivity and embodiment. Our goal is to expose the gendered and embodied intra-actions by which drugs and drug effects are co-constituted in human and nonhuman relations. Rather than treat these effects as stable and discrete, we aim to reveal the ways drugs and bodies, in their intra-actions, are transformed while constituting new subjectivities. Our analysis discusses three key themes: *movement*, *ambivalence*, and *sociality* in disclosing some of the key intra-actions by which this co-constitution transpires. Analysis of these key themes will help to reframe discussion of the subjective effects of medication in terms of complex entanglements*.* We follow Barad's (2007, p. ix) conceptualisation of entanglement as more than ‘to be intertwined with another’, instead considering how ‘individuals emerge through and as part of their entangled intra-relating’ (see Fitzgerald and Callard, 2016). We unpack the entanglements of affects, subjectivity and matter, rather than the more common focus on objective measures of treatment efficacy predominantly found in biomedical literature (Angell and Bolden, 2015). We close by briefly considering the implications of this reframing for ongoing discussions of the governance of conditions attracting psychiatric diagnoses, and the experience of this governance.

## 2 Theoretical framework

### 2.1 ‘Disease-centred’ logics, ontological instability and the production of ‘adverse effects’

Our research interests are set within the context of ongoing clinical discussions of psychotropic medication and their side effects that have focused almost exclusively on rates of compliance (e.g. Lambert et al., 2004), how to measure side effects (e.g. Waddell and Taylor, 2008), or that locate side effects as occurring in specific parts of the body, for example, weight (Zimmermann et al., 2003) and hormones (Bou Khalil and Richa, 2011). Such approaches to side effects have been termed the ‘disease-centred’ model of psychotropic drugs (Moncrieff, 2013, 2009) that necessarily (and, we will posit, somewhat erroneously) separates *therapeutic* effects from *side* effects, and treats symptomatologies of mental illness, therapeutic and side effects as discrete events. This approach inevitably installs ontological distinctions between disease and treatment effects, and between bodies marked by prevailing categories of social difference; assumptions worthy of considerable scrutiny which feminist science studies, and Barad's work more directly, so ably avails. In contrast, a disease-centred approach presumes that medication acts on the biological or neural underpinnings of mental illness. As a critical alternative to these assumptions, Joanna Moncrieff's work (2009, 2013) repositions the mechanisms of psychotropic action as ‘drug-centred’ – a model which encourages a more substantial engagement with the ensemble of effects that drugs can have. Through this lens, the actions of psychotropic medications can be approached in a ‘much more ambivalent light’ and the effects not ‘be parcelled off into therapeutic or adverse effects as if these were distinct’ (Moncrieff, 2009, p. 18). This insight will drive much of the analysis that follows, deployed through Barad's discussion of ‘intra-action’ and the co-constitutions of drugs and bodies in tangled relations of affect and force.

Other scholars have also sought to problematise the disease-centred model of psychotropic action by intervening in debates pertaining to the efficacy of drug treatments, the influence of ‘Big Pharma’ on the field of psychiatry, and the social and psychological harms of psychotropic medication (e.g., Choudhury et al., 2015; Jenkins, 2011, 2015; Pilgrim et al., 2011; Liebert and Gavey, 2009; Healy, 2004). Such work, as well as broader scholarship within the social sciences, has increasingly revealed the ontological instability of divisions between things; between clinical category and personal accounts; between ‘abnormality’ and side effects of achieving ‘normality’. For example, drawing on an ethnography with people who have experienced psychiatric care and psychotropic medication, several studies have unpacked the complexities of decision-making, often highlighting the role of side effects, negotiations and relations of power (e.g., Knight et al., 2018; Angell and Bolden, 2015; Seale et al., 2007, 2006; Rogers et al., 1998). In addition, concepts of phenomenology and embodiment have been harnessed to shed light on the complex effects of drugs (e.g., Morrison et al., 2015; McCann and Clark, 2004; Parnas and Handest, 2003; Usher, 2001). Usher (2001, p. 148), in a study on schizophrenia, for instance, observes that bodies lose their ‘taken-for-grantedness’ when people experience and learn to live with side effects of medication, while Morrison et al. (2015), in a study documenting side effects of antipsychotics, note that although participants doubted the benefits of their prescribed drugs, they were resigned to enduring often-debilitating side effects. This work has highlighted the difficulties of assuming clear lines between symptomatology, side effects and illness experiences, and the importance of phenomenological studies in revealing the resultant complexities for decision-making related to drugs and health care. They also point to the need for more holistic methodologies capable of capturing the full range of medication and treatment effects, particularly in terms of interactions and interferences between these effects (Duff, 2013). Work in feminist science studies on the social, affective and material entanglements of bodies and technologies (Fraser et al., 2014; Roberts, 2014; Vitellone, 2011; Barad, 2007; Waldby, 1999) offers some important insight into rethinking the effects of drugs.

### 2.2 Entanglements, iatrogenesis and ‘revealing’ side effects

Whilst the disease-centred approach to psychiatric care tends to delineate between ‘things’, we argue that the effects of and reactions to medication always emerge within intricate material and affective entanglements. The term ‘iatrogenesis’ is often used to refer to ‘adverse effects’ experienced during medical intervention (Waldby, 1999). However, this is not to suggest a simplified model of ‘harm’ whereby distress can be ascribed to the drug alone or to psychiatric institutions. As Catherine Waldby (1999, p. 79), writes ‘any biotechnical intervention inscribes itself into a complex, dynamic of corporeal animation and relationship, which redistributes its intended effects according to its own shifting logics’. Thus, iatrogenic effects disrupt cause and effect narratives of drug functioning, and unsettle the epistemology of the drug itself. As medication traverses through the body, it is broken down and absorbed in blood and organs, becoming further entangled in one's subjectivity. Researchers with personal experiences of mental distress have emphasised the need for research on psychotropic medication to investigate and acknowledge how ‘these substances … not only affect our bodies and minds – they enter our lives and shape our biographies’ (Russo, 2018, p. 16). In addition, such entanglements often do not ‘appear’ (in clinical conceptualisations of symptomatology, side effects and therapeutic effectiveness) only as the medication is swallowed, insofar as knowledge of drugs is always, already implicated in understandings of mental health and subjectivity that precede and give meaning to this ingestion. Entangled in discourses and social practices, medications are perhaps better conceived as *assemblages* of social, affective and material forces that affects bodies in complex ways, as they affect subjectivities (Duff, 2014).

Such articulations of encounters with drugs, and the assemblages in which these encounters are sustained, rely upon an understanding of institutions, medication and individuals as *mutually-constituted* and *entangled*; what Barad (2007) has termed ‘intra-action’. In contrast to ‘interaction’, intra-action recognises that agents (here: psychiatry, psychiatric patients, drugs, institutions) do not precede their encounters with each other; they ‘emerge through’ intra-action and are always co-constituted (Barad, 2007, p. 33). Material objects, including psychotropic medication, cannot be considered stable entities with specific properties which act *on* independent body parts. They become particular things in relation to other things, forces and bodies (e.g., toxic, therapeutic, enabling, side effect producing and disease preventing). Barad also considers that humans (and human actions) need to be approached in a similar manner. Therefore, how people are ‘prescribed’, take and engage with psychotropic drugs require a re-evaluation that considers the various entanglements of body, mind, discourse, institutions and wider society that inevitably shape consumption events.

How might this type of approach be applied in this empirical context? What are the intersections (and routine assemblages) of affect, bodies, expertise and pharmaceuticals, and what interests do these service? Extending Barad's work, Elizabeth A. Wilson reconceptualises depression as entanglements of moods and medications, describing the work of selective serotonin reuptake inhibitors (SSRIs) as sets of ‘intra-actions’. For Wilson (2011, p. 286), writing on the ‘side effect’ of suicidal ideation and SSRIs, this proposition suggests that ‘Suicidal ideation is not an isolated, delimited cognitive event; it is the disequilibrium of a neuro-pharmaco-ideo-affect system given voice’. Barad, Wilson and others (e.g., Roberts, 2014) provide a language for examining the entanglements of psychotropic medication, mental illness and subjectivity that will guide the empirical analysis to follow.

The three (entangled) thematic axes developed in this paper demonstrate how psychotropic drugs are *productive* of subjectivity *through* their promises and potential, their unexpected harms and the institutions from which they are inseparable. In developing these themes, we are presented with a system of entanglements from which isolated side effects cannot be excavated. Our paper will demonstrate how the *work* of pharmaceuticals (necessarily done in relation to other actors/actants) is inherently ambivalent and paradoxical, infused with conflicting relations between recipients and diffuse effects. Treatment in the form of psychotropic medication and their effects, produce complex affiliations between subjective hopes for recovery, potential therapeutic value and the possibility for disappointment or even harm, and the alleged promises of the drug (Rose, 2007).

## 3 The study

This paper focuses on the narratives of 29 people diagnosed with a mental illness in the Australian mental healthcare system (this study is described further in Knight et al., 2018). Participants were recruited between February and December 2014, via advertising through mental health community support services, newsletters and online. Advertisements briefly detailed the aims of the study and researchers' contact details. This open form of recruitment provided the widest possible reach in the community. Following contact via email or telephone, prospective participants were provided a ‘plain language’ statement explaining the study in detail. Participants were advised to contact researchers for further questions. Eighteen women and eleven men aged between 22 and 65 years old were interviewed. Whilst most were Australian-born, participants reported diverse ethnic backgrounds including Aboriginal Australian, Punjabi Indian, Greek/Egyptian, Maltese, Italian, Australian-Brazilian, Portuguese, British and Irish. Participants' psychiatric diagnoses were self-reported, most had received more than one diagnosis during their encounters with mental health services, with psychosis, bipolar disorder, schizophrenia and major depression most frequently disclosed. Additionally, most participants were on psychotropic medications at the time of the interview and reported extensive histories of taking various drugs. Many participants had experienced involuntary treatment (community treatment order or involuntary hospitalisation).

On the day of the interview, the study was discussed with participants, and they provided informed, written consent. Participants were advised they could stop the interview at any time and/or withdraw consent to participate in the study. The second author was the lead investigator on this interdisciplinary study and the fourth author was a member of the project's advisory group. Ethics approval was granted by the Monash University Human Research Ethics Committee (CF 13/2980–2013001607).

The interviews from which data for this article are drawn were collected by inviting participants to firstly provide unstructured accounts of their experiences of being diagnosed with mental illness. Participants could begin their stories at any point in time, with some choosing to start with first clinical encounters of diagnosis, and others earlier in childhood, thus recollecting early experiences of symptoms associated with mental illness. Following this initial narrative, participants were asked to further clarify encounters with health professionals, instances of voluntary and involuntary hospitalisation, relationships with friends and family, experiences of treatment (pharmaceuticals or psychotherapies) and ideas on personal recovery. This enabled us to identify congruities and divergences in how concepts such as subjectivity, agency and illness appeared in different contexts. Interviews lasted between one to 3 h and were conducted in a place comfortable for the participant. The interviews were audio or video recorded and transcribed verbatim. Transcripts were returned to participants to review and provide them with an opportunity to, upon reflection, remove sections of the interview text if they wished.

## 4 Analysis

Transcripts were analysed in a series of iterative steps. Initially, they were read closely by the two first authors and discussed broadly in the light of existing literature. We used participants transcripts both ‘as a rich source of qualitative data’ and to observe how individual stories moved in and out of the trajectory of collective narrations (Stone and Kokanović, 2016, p. 101). This approach directed our attention to ‘nodes of interest’ that warranted further critical attention and could alert us to concealed meanings (see Davidsen, 2013). Emergent themes were noted, following by generating overarching themes and selection of illustrative quotes to create a first draft of the paper. Other authors joined the writing process, contributing to structure, refining the analysis and the identification of additional relevant literature. Subsequently, further drafts were created collaboratively. The theoretical framework of feminist science studies enabled us to conduct data analysis and writing processes as emerging, entangled phenomena. Attending to how data is read and ‘emerges’ through authors' practices, we approached our analysis as assemblages of subjective realities and knowledge production (Jackson and Mazzei, 2012).

As noted, our analysis was informed by feminist sciences studies (Fraser et al., 2014; Vitellone, 2011; Wilson, 2011; Waldby, 1999), particularly the work of Karen Barad (2007), to demonstrate the complex ‘intra-actions’ by which treatment effects, subjectivities and embodied experience are co-constituted in complex social, affective and material relations, with related attention to how discourses shaped participants narratives (Jackson and Mazzei, 2012). Our analysis extends previous qualitative studies of experiences of taking psychotropic drugs (e.g., Morrison et al., 2015) and contributes to debates rethinking the action of drugs (Fraser et al., 2014; Trivelli, 2014; Wilson, 2011) by considering the role of objects, actions and institutions in the constitution of subjectivity. While we aimed for cultural diversity in the participants, and people reporting diverse ethnic backgrounds were interviewed, no participant departed from ‘Western’ ontologies regarding drugs.

This analysis and its theoretical framings point to the impossibility of isolating unwanted side effects from the totality of experience of medication and their effects situated in the context of people's lives. The experience of consuming psychotropic medication inevitably entails highly variable, complex and inter-dependent effects and outcomes. This complexity is manifest in participants' reports of movement, ambivalence and sociality, and the ways medications are generative of subjective feelings of hope, value, change and fear. We commence with a discussion of movement and its varied physical and subjective iterations.

### 4.1 Movement

#### 4.1.1 Physical motion

One of the dominant distressing effects of psychotropic medication reported by participants pertained to alterations in the way their body feels, looks and moves. A compelling example offered by participants is the profound disorientated movements known as akathisia. Akathisia has been defined as ‘subjective’ feelings of ‘inner restlessness and the urge to move’ and ‘objective components’ such as ‘rocking while standing or sitting, lifting feet as if marching on the spot’ (Miller and Fleischhacker, 2000, p. 73). Van Putten (1975, p. 43) notes that akathisia is an affective state, a desire to move, not a ‘pattern of movement’, and adds it is ‘mistaken’ to consider akathisia an ‘exacerbation of the original mental illness’. However, we argue, in light of the accounts detailed below, that such conceptualisations obscure the entanglements of subjectivity, embodiment, affect and medication, and render ‘mental illness’ as an objective, neutral and ahistorical entity in defiance of the lived embodiment of illness and treatment.

Eamon, a 35-year-old man diagnosed with schizoaffective disorder, offered an account of akathisia while on trifluoperazine: ‘I was running to places … one day I ran so far … and I thought, where am I? You know, I had to get home’. The effects Eamon experienced caused him to move incessantly, he described running and ‘[pacing] for a long time’. Eamon's descriptions of drug effects demonstrate the ‘intra-action’ of medication, effects, embodiment and subjectivity by revealing how each is co-constituted in the experience of consumption. This finding is in line with Barad (2007) concept of ‘intra-action’, which we found useful in analysing how Eamon's corporeal experience of medication was produced concurrently with changes in this experience. For Eamon, drug effects did not follow a simple linear arc in which consumption at time ‘point a’ led predictably to effects at time ‘point b’. Rather an intra-action transpired by which the drug and its effects were co-constituted as Eamon's corporeal and subjective experiences were modified. His affective experience is marked as ‘compulsive’, defined by an urge to move, while he described his desire to be still as frustrated by the estranged embodiment of perpetual motion. We would suggest that this manifestation of akathisia signals the slippery qualities of symptoms of mental illness where subjectivity, embodiment, medication and mental illness are intra-actively produced. It is essential to think of the relationship between psychotropic medication, the body and psychiatric diagnoses as unsettled *and* inextricable. Beyond questions of continual movements and their role in producing subjectivity, akathisia also raises questions of how subjectivity can be approached as sets of connections to objects, bodies and *motion* itself.

In contrast to continual movement, Nick, a 41-year-old man diagnosed with schizoaffective disorder described taking several typical antipsychotics (see Moncrieff, 2013) that made him ‘dystonic’: ‘It made me not being able to move properly. I was not in a very good shape, and I was having muscle spasms … my arms were stiff. I was like an ironing board.’ Nick also expressed frustration at the advice he received from his psychiatrist, ‘he didn't change the medication. He wasn't looking at me as a person. He was looking at me with symptoms of a mental illness'. We see an interesting difference in the interpretations of side effects between Nick and his psychiatrist. In Nick's account, the medication is bound up in a ‘dystonic’ embodiment which distresses him. The effects of medications here cannot be fully distinguished from the therapeutic promises of the drugs. The psychotropic medication is seen to animate material-discursive relations to drugs. The actions of drug, subjectivity and institutional power relations manifest in the response of Nick's psychiatrist who parcels off the ‘side’ effects and thinks of ways to minimise them, ignoring the embodied, material-discursive entanglements of Nick's subjectivity with the drug. For Barad (2007, p. 91), ‘practices of knowing are specific material engagements that participate in (re)configuring the world’. Relations of power, such as between psychiatrist and patient, do not simply create positions of subordination and domination in the world. The materiality of psychotropic drugs and their inextricability from institutions and individuals, inevitably reveals relations of power, sociality and affect that actively reconfigure how knowledge is produced.

#### 4.1.2 Gendered cellular motion

All participants who were taking psychotropic medication reported experiencing a range of effects embodying the therapeutic regime, many of which were referred to as debilitating. Medication fractured the physiological order to which participants were accustomed, with significant ongoing impacts on subjectivity. For example, Alejandra, a 33-year-old woman with a diagnosis of schizophrenia, described how risperidone caused changes to her body:

I wasn't menstruating … So that was quite upsetting … I started lactating … I shouldn't be on medication that's making me lactate … being forced to be on [risperidone] which you know causes lactation … I think it was a bit cruel.

Interestingly, a 40-year-old male participant with a diagnosis of bipolar affective disorder I, Vincenzo, experienced shock when he learned that he could begin to lactate. He could not recall the drug's name, but this possibility disrupted his ideas of male embodiment:

one of them that really kind of freaked me out … was the fact that as a male I could start lactating … I was like this … is going to like damage me big time if that's something that's going to happen from taking these tablets.

Another participant, Amrick, a 32-year-old man who was diagnosed with paranoid schizophrenia, did not use the term ‘lactation’ but explained that one side effect of taking quetiapine was an increase in his prolactin levels and an enlarged breast area. Combined with impotence, these corporeal shifts caused him significant distress.

Feeling distress because of the absence of menstrual periods reveals the troubling of gendered identities through new (and disruptive) pharmaco-physical interactions, reflecting the entanglements of medicalisation, identity, and ‘normality’. Alejandra *expects* the manifestation of menstrual periods. By the same token, her body lactating at a time when it is neither expected nor ‘needed’, caused significant concern such that she demanded a change of medication. The cultivation and disruption of gendered subjectivities are also clearly at work in Alejandra, Vincenzo and Amrick's experiences. Beyond disturbing gendered physiological form, gender is produced as problematic through the (potential) encounter with breast milk and sexual dysfunction. It is thus not simply a question of drugs encountering gender, but one of drugs and gender being mutually constituted as the medication encounters bodies, transforming subjectivities and disrupting identities.

A mouth that drools, a breast suddenly lactating, a body part distended, tics in a section of one's visage, these complex effects of psychotropic medication transform embodied subjectivities, sometimes quite abruptly. The overall effects of drugs fragment and ‘re-assemble’ the body in different ways. These changes, quite paradoxically, become indicators of illness upon the body; they metaphorically *bring the symptoms to the surface*. Therapeutic regimes, in their embodied effects, function to reveal (often previously concealed) features of the person and affliction. Or do they produce these very symtomatologies? It is here that Barad (1998) work on the *before, during* and *after* come into play; the symptoms follow the treatment, or does the treatment produce the symptoms? These accounts challenge the linear progression of time and therapy, that is, from emergence, treatment and recovery, to illustrate the unsettling of temporal delineations between symptom, side effect and ‘normality’.

The implication is that various assumed ontological splits (person/population, drugs/disease, recovery/health, illness/symptoms), slip into murkier territory. The appearance of illness is thus more accurately viewed as a co-production of this particular scene *and* the person, rather than mere manifestation; it is induced emergence, mediated by pharmacological materials and respective expertise, rather than physiological manifestation. ‘Individuals’, ‘symptoms’, ‘afflictions’, ‘recoveries’ become collective and relational productions (with agreements and disagreements therein) rather than reflecting taken-for-granted ideas about individual diseases (symptoms) and collective responses (therapies). Such distinctions become untenable, and in doing and being so, raise questions about why one would assume such things hold together at all. This speaks to the power of an individualised logic in the collective imagination, and the need for ordering in the context of disordered being in the world. Thus, it is not that symptoms follow treatment or treatment produces symptoms, it is that there is no meaningful, coherent line between these concepts. And that the attempt to delineate is an act of power and in turn concealment of the pernicious effects of pharmacological interventions (which can become packaged with disease).

#### 4.1.3 Resisting motion

For a few participants, the enmeshment of mental illness and distressing effects of medications caused disruptions to subjectivity (and life) to the extent that they decided to cease their medication. For instance, Florence, a 37-year-old woman diagnosed with reactive psychosis, noted that the experience of side effects, which in her narrative is enmeshed with the experience of mental illness, stood in stark contrast with what life in her thirties should be. As she said, ‘[this] is not the life’. The combination of constant fatigue and weight gain seemed to fragment Florence's understanding of who she is: ‘I was putting on weight … it was still enough for me to feel out of touch with myself’. Florence described her thoughts as ‘paranoid and conspiracy like’ but maintained she never heard voices. After being given diazepam and another medication which name she could not recall, Florence also explained that she heard ‘an audio voice [she] never heard before in [her] head saying two times, “Shut up, shut up”.’ Her symptoms were exacerbated by the medication, and she was unsure whether she had been given an additional drug. Her account reveals the ambiguous qualities of drugs, and how drugs can become continuous in accounts of mental illness. Florence's subjectivity as a person diagnosed with a mental illness, and her own understanding of her condition, are further transformed through contact with drugs.

### 4.2 Ambivalence

All participants taking psychotropic drugs expressed complex ambivalence when explaining the diversity of effects they experienced. This ambivalence pertained, on the one hand, to feelings that medication was effective in alleviating certain symptoms even as it caused distressing adverse effects. On the other hand, ambivalence manifested in expressions of doubt as to whether the drugs were working at all. Participants who take drugs are necessarily shadowed by the impossibility of knowing what might have happened – to their health, to their symptoms – if they had not taken medication. If additionally, there is doubt over whether the medication works, there is also the difficulty of determining whether the medication is *inducing* new symptoms, as well as, or instead of, potentially exacerbating – rather than allying – existing ones. Such epistemological uncertainty – both about how to interpret the action of drugs, and how to judge one's own medicated life in relation to an imagined life that might have not included drug-taking – was prominent in several interviews. Indeed, while clinical discourses of psychotropic medication identify the labour of drugs on specific neurons and organs (Keltner and Folks, 2005), experiential narratives reveal the impossibility of firmly locating the effects of drugs, while questioning what drugs are ‘supposed’ to do for the individual. For Seamus, a 39-year-old man diagnosed with bipolar II and depression, the effects of the drugs were difficult to specifically locate:

I've often wondered whether I've had any benefit from medication I've taken … there's no way that my psychiatrist is going to stop prescribing me medication … at times I've felt better about being on the medication … it hasn't stopped me from becoming unwell, ever … is medication supposed to do that? I don't know.

Seamus' account reveals doubt about the efficacy of medication, the accuracy of diagnosis and the purpose of treatment. Similarly, Molly, a 47-year-old woman with several diagnoses over time including borderline personality disorder, major depression and rapid-cycling bipolar disorder, considered that while the ‘only thing [she gets] are side effects’, she continues her treatment because of her psychiatrist suggesting she will ‘be worse’ without medication and her concern that she would ‘be seen as non-compliant’.

In Molly's and Seamus' encounters with psychiatric diagnosis and medication, subjectivity emerges in deep enmeshment with institutions, psychiatrists, mental illness and the effects of medications. Such encounters draw our attention to the entangled intra-active processes through which subjectivities are produced. Take the subjective identity of the ‘non-compliant’ patient that Molly is so concerned about; this identity does not pre-exist the body to which it becomes attached, but instead is co-constituted in its entanglements with health practitioners, treatment guidelines, mental health policy, clinical settings and practices. Molly is prepared to tolerate ‘wanting to vomit every morning’ in order to avoid this subjectivation, and her corresponding identification as a non-compliant subject, in ways that remind us of Barad's (1998, p. 106) argument that ‘“subjects” and “objects” do not pre-exist as such, but are constituted within particular practices’. Molly, the ‘compliant subject’, is one example.

Other participants' experiences were also marked by ambivalence, representing the ‘multiple paradoxes of lived experience’ (Jenkins, 2015, p. 70). Diagnosed with major depression and bipolar II, 37-year-old Keith noted: the medications ‘might have been stopping me from hurting myself but … they weren't helping me really in terms of leading a life and I was totally at the mercy of the side effects.’ We return to the question of what kind of life participants imagined for themselves through medication in the following pages. For the time being, we would like to suggest that more than a deep ambivalence to medication, Keith's experience also reveals that the effects of medications are not symmetrical across bodies and lives. For Keith, medication effects are utterly entangled in complex relations between his subjective experience of desires for self-harm, hopes for a particular kind of life, and frustrations at living at the ‘mercy’ of unpredictable effects.

Psychotropic drugs can be experienced simultaneously as ‘saviours’ bringing quietude and impediments causing distress, indicating how subjectivity is transformed through the ambivalences of experiencing the asymmetrical and complex effects of the pharmacological regimen. As agents and voices of resistance and negotiation within the field of psychiatric knowledge, our participants considered trust and control paramount to their treatment. Psychotropic medication became a signifier of both medical expertise and a loss of control. Medication became symbolic of a system working to withdraw agency *and* improve symptoms and quality of life. Larry, a 37-year-old man with multiple diagnoses including schizoaffective disorder and obsessive compulsive disorder, shared his thoughts on how he lives with the ambiguities of his diagnoses and psychotropic medication:

it's got side effects and that's unpleasant … You have to put up with them. There's no other word for it. … unless some miracle cure comes … this is going to be my life for the rest of my life … it's like having an incurable disease. It can be managed it need'nt get in your way all the time … But it's never going to quite leave your consciousness. And I think you can run away from it every now and then. But you just have to realise that you just have to take your medication at 10–10:30 at night as usual.

Larry's evocative words – that psychiatric diagnoses *never quite disappear* from one's consciousness – stand in stark contrast to discourses of mental illness which rely on the ‘master clinical trope’ (Jenkins, 2015, p. 38) of chemical imbalance. For Jenkins (2015, p. 39), the language of chemical imbalance does not capture the complex subjectivity at work through symptoms of mental illness and the ingesting of psychotropic medication. Subjectivity, in Larry's example, is deeply enmeshed in diagnoses, and how drugs work and do not work, entailing a ‘becoming-with the drugs’ (Trivelli, 2014, p. 159). The drug does not merely materialise its effects (therapeutic or otherwise) as it dissolves and journeys through the body, nor does it remain a passive object prior to swallowing. The psychotropic medication can be approached as *already doing*, involved in the task, in a network of humans and objects. In our example, the doing of the psychotropic medication is also invested with promises, hope and meaning. Thus, mental illness never leaves individuals' ‘consciousness’ and is inseparable from medication that needs to be taken ‘at 10–10:30 at night’ and its ensemble of effects. Instead, we can see how mind-bodies-drugs are mutually constituted and inextricable from each other's entanglements.

#### 4.2.1 Institutional power relations and the mutability of drugs

Ambivalence about medications and treatment for mental illness mean that adverse effects cannot be simply located in specific parts of the body and documented as though they are separate from institutional power relations. Alejandra, for example, spoke of the lack of trust in her ability to take medication:

Rather than trusting that I would take the medication if I was given the choice of oral … tablets but they thought that it was best to keep me on the [risperidone] injections so that … I wouldn't lie … I found they were just taking control out of … my life and I had no power in the process.

This account of the transition from oral tablets to injections provides insight into the ‘social life’ of drugs (Duff, 2013) and the mutable qualities of medications. In Alejandra's account, psychotropic medications are neither stable entities nor predictable objects. Not only can their shape change, they are also *in the process of transformation* when in contact with certain subjects. Instead of viewing the syringe as a ‘dead device’, Vitellone (2011, p. 201) encourages us to view it as ‘fully alive to the event at hand’ (see Fraser et al., 2004). By conceiving of objects as enactments, Barad is able to unpack the agency of objects involved in scientific experiments. For Barad (2007, p. 203), the material participation of scientists and their apparatuses is central to the production of knowledge. Thus, if we consider the syringe as apparatus, it becomes possible to view Alejandra's subjectivity as entangled in the materiality of the needle and the mixture held within. The encounter not only withdraws power from Alejandra, as the ‘non-compliant’ subject is enacted and entangled, her subjectivity is actively undone and remade through those discourses as well as the syringe itself, which is loaded with meaning and knowledge.

If the concept of ‘intra-action’ signifies that the object studied cannot be separated from the practice and knowledge that makes it comprehensible to us, then psychotropic medication needs to be reimagined too and re-examined through its ensemble of effects, the discourses of hope attached to the drug, and the institutional power relations that shade every encounter between bodies and medications. In an interesting example of the mutability of objects described by Barad (1998, 2007), 56-year-old Alexis diagnosed with schizophrenia, considers medication as food: ‘I take an orange and it makes me feel better and if I take … a tablet that makes me feel better, what's the difference between an orange and the tablet?’ Referring to medication as ‘mind food’ (Ecks, 2014), Alexis believes there should not be any stigma attached to drugs. He also introduces pharmaceutical corporations to the complex relations of power entangled in drugs:

a lot of energy has been placed, a lot of research, a lot of … getting chemicals together … to put that compound together in that tablet form. A lot of energy has been put in developing that medication for the wellness of the person … [this effort] should be respected

Knowledge of the drug (whether cultural, clinical, experiential or otherwise) is crucial to what makes it ‘work’; a drug *becomes*, rather than *is*, and its subjects are co-producers of its effects. One cannot detach the drug from its dispersed effects on bodies (organs, neurons, blood and so on), how the effects are *known* and *narrated* and by whom, and the institutions and persons that make the ingestion of drugs possible (psychiatrists, hospitals and pharmaceutical corporations). Beyond the object itself, and its complex entanglements in processes of treatment, care and support, our analysis revealed key links between the expressions of subjective identity in the consideration of medication effects, and the character and experience of sociality.

### 4.3 Sociality

Apart from their ensemble of effects on the brain and other parts of the body, a key aim of psychotropic medication is to facilitate the subject's return to a functional, normative social life. In other words, drugs are commonly said to help individuals become reacquainted to ‘normal’ sociality. This return to the social or functional involves navigating competing economic, relational and intimate demands, such as family and work. Qualitative and ethnographic studies with people taking psychotropic medication have tended to render the pill as a ‘static’ object, even while acknowledging and unpacking its therapeutic and adverse effects (e.g., Morrison et al., 2015; Steffenak et al., 2015; Carrick et al., 2004). This approach has not always been alert to the social life of medications, and the ways their ingestion transforms the networks in which drugs are embedded. The narratives of our participants not only demonstrate how psychotropic medications have a social life, they also reveal a kind of *pharmaco-affective sociality*. Such sociality, deploying Barad's insights, does not distinguish between *the subject* that swallows *the object* and engages in social life. Instead, the medication enacts as it participates in forms of pharmaco-affective sociality whereby the drug can ‘lubricate’ social contact and transform subjectivity as an ‘always unfinished, interrupted, intricate and nonlinear’ project (Kokanović and Flore, 2017, p. 331).

In his discussion of the competing demands of his life, Amrick explained that one must prioritise medication, rather than finance or family:

They'd just go … have this medication … your first priority is in your medication. There's other priorities, you know. If it's not accommodation it's your finances, if it's not your finances you're worried about your family … So until I got it through my head that my medication is the most important thing to keep a … stable mind.

Amrick's comments evoke the need to ‘spend time’ *on* and *with* medication. We can also see the entanglements of illness and medication as priorities demonstrating how understandings of mental illness are further transformed through contact with drugs. The labour of taking one's medication requires more than ‘responsibilised’ subjectivity (Rose, 2007), it also requires *attunement* to pharmaco-affective sociality.

Scholars researching experiences of taking psychotropic medications have noted the role of optimism and the ‘future’ in people's narratives (Carrick et al., 2004). Our analysis however complicates the idea that taking medication simply includes dimensions of hope (for recovery, for example). While we acknowledge that hope plays a key role, we draw on Barad (2007) to suggest that concepts of futurity, drugs, subjectivity, and sociality itself, are made and remade in their intra-actions with each other, another reason why the theme of ambivalence runs so strongly through our participant's accounts. Thus, the agency that humans are often assumed to ‘possess’ when articulating ideas of the future, ‘*is an enactment, not something that someone or something has*’ (Barad, 2007, p. 235). It therefore becomes possible to illuminate how accounts of taking psychotropic medication participate in ‘making’ particular kinds of lives characterised by ambivalent experiences of hope and fear, promise and stasis.

Several participants spoke of hopes for their lives, and the role that medication plays in this configuration of hope as an affective and material orientation to an imagined future self. Wendy, for example, a 49-year-old woman with multiple diagnoses including schizophrenia and bipolar disorder spoke of her desire to ‘go off the meds’, even as she acknowledged, ‘I've been off them for a period but they put me back on so I must need them’. Meanwhile, Cheryl, a 40-year-old woman diagnosed with schizophrenia believed that the medication was ‘good enough’ if it ‘gets rid of the experiences … of hearing someone talk’. Some also referenced how medications might mediate relations to family and friends in the future, depending on the material and social effects of these medications. Discussing her medication, Joan, a 36-year-old woman with a diagnosis of bipolar II, described her return to sociality, which she attributed partly to the medication, as problematic because of her desire to have children:

The biggest hurdle for me now is that I'm at the point where I am happy with my life and job and study … my partner and I would like to have kids. But that involves coming off medications and that's quite nerve-wracking.

While medication may have facilitated her return to ‘life and job and study’, they are now positioned as obstacles to Joan's progress in social and intimate life. A degree of optimism *through* medication circulates in these narratives reflecting the relational subjectivities at work in them, as do notes of fear and ambivalence.

For several participants, drugs, in perturbing emotions, produced a different dimension of pharmaco-affective sociality whereby individuals' capacity to affect and be affected was disturbed and reconfigured. A capacity for the expression of feelings was viewed as a therapeutic activity that some participants could not access. Riya, a 22-year-old woman whose diagnoses included borderline personality disorder, referred to the metaphor of the zombie when conveying her emotions while on quetiapine: ‘I felt like I'd lost my body … I was just zombified all the time’. Such images served to make experiences of pharmaco-affective subjectivity more intelligible for the interviewer, and to communicate the profound impact of the medication on participant's ability to *feel* and manifest emotions*.* After changing her medication Alejandra, for example, touched her heart and expressed relief: ‘I feel a lot more emotional as a person but not to the point where I'm unwell but I just feel like I'm feeling emotions a bit more’.

Treatment through psychotropic drug therapy is believed to provide options and hope for a manageable social life, if not ‘recovery’ from symptoms associated with mental illness. The valuing of one kind of life over another emerged as a complicated prospect for our participants. Indeed, while pharmaceutical therapy for mental illness may indicate that a life of hearing voices, for example, is not desirable, so too may be a life on psychotropic medication. There is a tension between the medication working both for *and* against people, alongside an overarching desire to live ‘medication free’. Joan expressed this entanglement of ambivalence and pharmaco-affective sociality as follows: ‘what works for one patient isn't going to work for the next patient … in an ideal world there'd be a magic pill that everyone could take the same things … and live a happy, fulfilling life.’ Sociality is a fundamental part of the dynamism of forces that constitute kinds of subjects in treatment, in the event of consuming particular medications (Duff, 2014).

## 5 Conclusion

In qualitative explorations of the experience of being diagnosed with mental illness, the complexities of mediating medications – as variously disabling, enabling, normative and liberative – has been an importance facet of trajectories toward survivorship, wellbeing and recovery (e.g., Morrison et al., 2015; Carrick et al., 2004). This paper has sought to add further nuance to this scholarly and empirical field, by considering the modes of subjectivity intra-actively produced through psychotropic medications. Understanding those modes of subjectivity, and the particular relations, practices and events in which they are produced, offer novel grounds for interrogating the character and meaning of side effects, affliction, wellbeing and recovery. Rather than insisting upon clear spatial and temporal distinctions between side effects and therapeutic effects, for instance, this offers an approach to being ‘relieved’ from symptoms of mental illness as non-linear, and messier and more complicated than often represented. The symptoms of mental illness and effects of medications have often strange temporal trajectories and complex ways of becoming visible/knowable to the person experiencing them. Our analysis clarified, then, how nuanced and heterogeneous are the adjudications that people make about the role that taking – or indeed not taking – psychotropic medications might have in relation to putative futures that *might* bring ‘health’. The intra-active approach has revealed forces of movement, ambivalence and forms of sociality that disturb the assumptions that underpin much of the mainstream literature on mental illness treatment. In so doing, we have sought to unpack some of the affective and material relations that people develop with (and in relation to) psychotropic drugs. This opens a critical space where ‘side effects’, ‘therapeutic effects’ and subjectivity – and the object of the drug – are intimately entangled and inextricable from each other. In terms of the social science of mental healthcare, the accounts presented here suggest that experiences of psychotropic medications are in fact better articulated as intra-subjective emergences, as co-produced and inflected by power relations. Further, that these relations evade temporal simplicity (i.e. thinking beyond *prior* drug effects or progressive/typical ‘symptoms’ to relieve) but rather a vitalistic process of becoming (rather than being) and one that operates on and through a brain-body-world axis (Blackman, 2012).

The particular importance of this work lies in highlighting the ontological problems associated with ‘typical’ ways of knowing and thinking about medications. For example, the assumptions around pathologies (things to *supress*) and necessary side effects (things to *tolerate*). The forms of ambivalence expressed point to the ontological instability of such ideas. Lines between symptomatic and side effects, functioning and dysfunctional, may be much murkier than often recognised. Hence, the idea of medication as entanglement – with body, person, authority, institution, power and identity – is more theoretically useful way into examining drug effects. It allows us to ask, what does this technology *do* in and for this person; what does the medication itself embody, perhaps a certain version of stability? A series of side effects deemed acceptable in the past by people in positions of authority to make such decisions? Other actors and subjects produce subjectivities in the present, and even future possibilities for recovery.

Pharmaceuticals and their effects, bodies, minds and moods form part of the dynamic system through which subjectivity is undone and remade. This is not to suggest the emergence of a coherent, ‘whole’ subject who overcomes illness and ‘lives with’ side effects as a kind of necessary harm. Rather, subjects are continually fragmented and re-articulated. The reality of the complex effects of medication, sensed, felt and narrated, reveals the contingencies and continual ‘becomings’, rather than stable entities, of medication and subjectivity. This suggests a new task in the treatments of experiences attracting psychiatric diagnoses requiring sensitivity to the ways subjectivities are contested, expressed and transformed in treatment, to sit alongside more conventional concerns for diagnosis, therapy and recovery. It also suggests that the experience of treatment and medication is not straightforward, and that any assertion of linear and predictable drug effects should be treated with caution. Instead, the ‘intra-actions’ of subjects, bodies, medications and treatment stand out.

## Funding

This project was funded by an Australian Research Council Linkage Project grant (LP130100557) and supported by partner organisations, including the Victorian Department of Health and Human Services, Mind Australia, Neami National, Wellways, Tandem Carers, the Victorian Mental Illness Awareness Council, and Healthdirect Australia.

Felicity Callard is supported by The Wellcome Trust (209513/Z/17/Z).
